# Single-cell transcriptomics provide insight into metastasis-related subsets of breast cancer

**DOI:** 10.1186/s13058-023-01728-y

**Published:** 2023-10-19

**Authors:** Shikun Zhu, Mi Zhang, Xuexue Liu, Qing Luo, Jiahong Zhou, Miao Song, Jia Feng, Jinbo Liu

**Affiliations:** Department of Laboratory Medicine, The Affiliated Hospital of Southwest Medical University, Sichuan Province Engineering Technology Research Center of Molecular Diagnosis of Clinical Diseases, Molecular Diagnosis of Clinical Diseases Key Laboratory of Luzhou, Sichuan, China

**Keywords:** Single-cell transcriptome sequencing, Breast cancer, metastasis

## Abstract

Breast cancer metastasis is a complex, multi-step process, with high cellular heterogeneity between primary and metastatic breast cancer, and more complex interactions between metastatic cancer cells and other cells in the tumor microenvironment. High-resolution single-cell transcriptome sequencing technology can visualize the heterogeneity of malignant and non-malignant cells in the tumor microenvironment in real time, especially combined with spatial transcriptome analysis, which can directly compare changes between different stages of metastatic samples. Therefore, this study takes single-cell analysis as the first perspective to deeply explore special or rare cell subpopulations related to breast cancer metastasis, systematically summarizes their functions, molecular features, and corresponding treatment strategies, which will contribute to accurately identify, understand, and target tumor metastasis-related driving events, provide a research basis for the mechanistic study of breast cancer metastasis, and provide new clues for its personalized precision treatment.

## Background

**Fig. 1:**
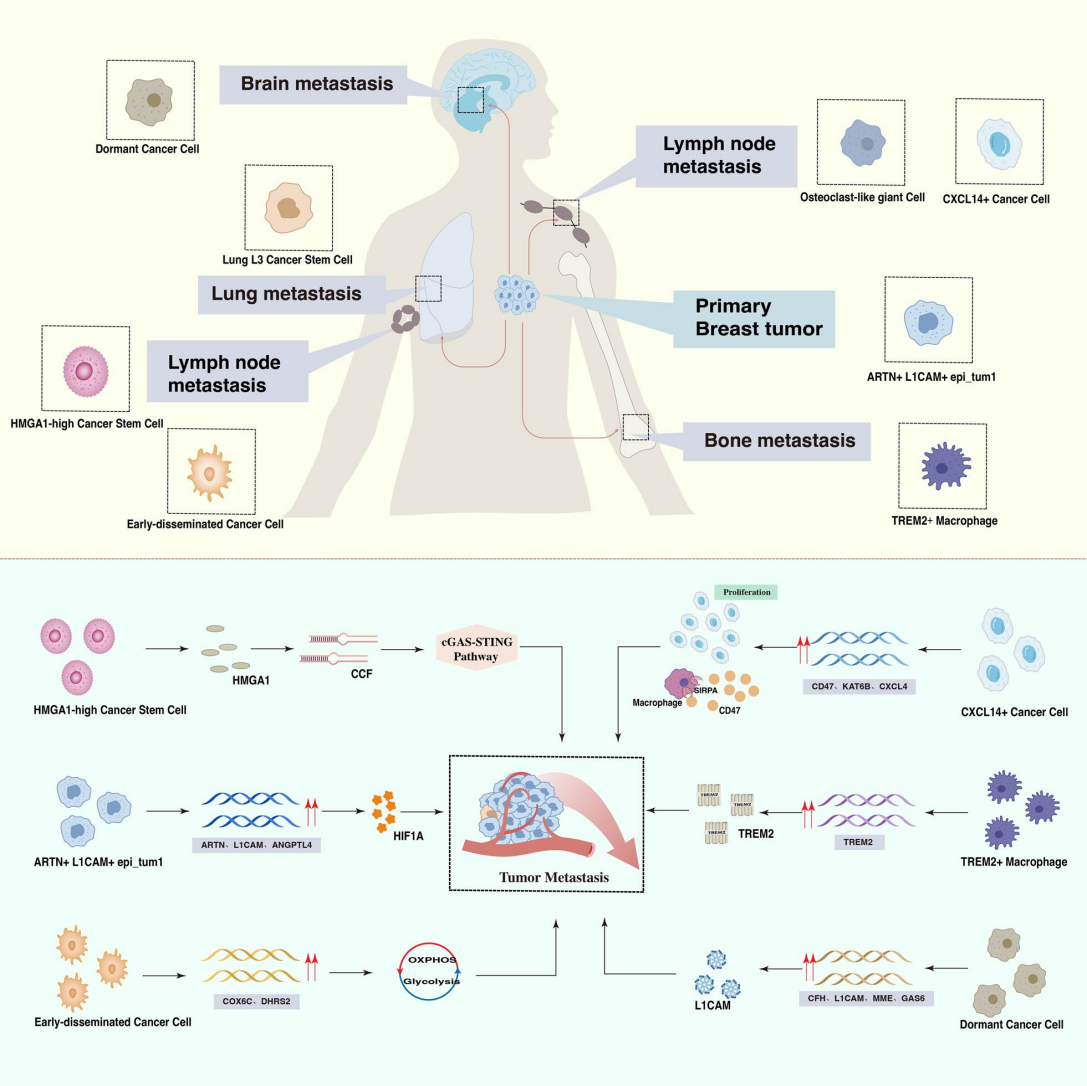
The above picture shows the tissues or organs where the various cell subpopulations are located, the following picture explains how cell subpopulations cause metastasis

Breast cancer (BC) is the most common cancer among women worldwide, and its incidence and mortality rates are increasing year by year. Although traditional treatments such as surgery and radiotherapy can effectively control the progression of primary tumors, BC metastasis remains the main cause of death in most cancer patients and poses a great challenge in clinical practice. Malignant tumors are characterized by their ability to spread from the primary site to adjacent or distant organs via the circulatory system or cavities, leading to tumor metastasis [1]. During tumor metastasis, cancer cells from the primary tumor spread to lymph nodes or the circulatory system and body cavities to distant organs. These tumor cells further colonize new sites, forming a new TME at the metastatic site. However, there are significant differences between metastatic TME and primary TME, which pose challenges to the study of tumor metastasis. The development of single-cell transcriptomics provides a more convenient and quick way to analyze the heterogeneity of the TME. Metastatic tumors have high sampling difficulty and heterogeneity. Still, the advantage of single-cell transcriptomics lies in the identification of rare cell types in highly heterogeneous tissues, gene feature analysis, and description of interactions between cells, which can infer potential differentiation trajectories to some extent, thereby solving the above-mentioned problems. Therefore, this review explores the special or rare subgroups related to BC metastasis and systematically summarizes their functions, molecular features, and corresponding treatment strategies, which will contribute to accurately identifying, understanding, and targeting events driving tumor metastasis, provide a research basis for exploring the mechanisms related to BC metastasis as well as the directions for personalized precision treatment (Fig. 1).

## Special cell subpopulations related to metastasis in the primary tissue of breast cancer

Cancer metastasis is a multi-step process involving an invasion-metastasis cascade, which starts from the primary tumor and develops into a new tumor colony in distant tissues. Primary tumor cells remodel and infiltrate the microenvironment and further spread to distant tissues, gradually developing into detectable metastatic lesions in these tissues. Therefore, the primary tumor is the starting point of all metastatic tumors and analysis of it can find early metastatic cancer cell subgroups, thereby helping us further understand the initiation process of BC metastasis.

Shi et al. [2] have discovered a group of hypoxic tumor cell subgroups related to metastasis (*ARTN*^+^ *L1CAM*^+^ epi_tum1) in the primary tumor tissue, speculated to be a potential new cell subgroup related to BC metastasis. Previous studies have confirmed that the marker gene *ARTN* is highly expressed in BC and can enhance the metastasis and invasion of estrogen receptor-positive (ER^+^) cells. The other marker gene L1CAM and its highly expressed gene *ANGPTL4* in *ARTN*^+^*L1CAM*^+^epi_tum1 have also been proven to accelerate the hematogenous metastasis of tumor cells from the breast to the lung under hypoxic conditions by up-regulating the expression of hypoxia-inducible factor 1 subunit alpha (*HIF1A*) [2–4]. Further analysis showed that hypoxia-related genes and pathways are up-regulated in *ARTN*^+^ *L1CAM*^+^epi_tum1, suggesting that it may be an important hypoxic subgroup in TME. Analysis of intercellular communication showed that this hypoxia-related tumor subgroup in the TME can accelerate the formation of abnormal blood vessels and tumor progression by up-regulating various related signaling pathways such as *ADM*, *VEGFA*, *EDN1*, and *ANGPTL4*. In addition, Shi et al. [2] have confirmed that the marker genes *ARTN* and *L1CAM* of the hypoxia-related tumor cell subgroup are potential prognostic indicators of poor prognosis in triple-negative breast cancer (TNBC) patients. In summary, this hypoxia-related tumor cell subgroup tends to overexpress genes related to angiogenesis and metastasis under hypoxic conditions, thus achieving self-renewal.

## Special cell subgroups related to metastasis in lymph node tissue of breast cancer

Draining lymph nodes and local lymphatic vessels are often invaded by malignant tumor cells at an early stage, and this process is influenced by the molecular expression characteristics and circulatory distribution characteristics of the TME [5]. Previous studies have confirmed that there are significant differences in genotype and phenotype between the tumor cells in metastatic lymph nodes and those in primary lesions. Metastatic tumor cells can colonize specific areas in the lymph nodes to form micrometastases, which can interact with various surrounding immune cells and induce lymph node matrix remodeling, thereby promoting the formation of an immunosuppressive microenvironment [6]. Therefore, lymph node metastasis is often considered a precursor to distant metastasis and an important determinant of the timing and scope of surgical intervention [7, 8]. With high-resolution single-cell sequencing technology, we can further map the immune atlas of lymph node metastasis, analyze its complex intercellular communication, and identify key regulatory factors affecting lymph node metastasis.

### Early dissemination-related tumor cell subgroup—early dissemination cancer cells (EDC)

Previous studies have confirmed that the malignant biological behavior of tumor cells at different stages accompanies significant metabolic shifts, which is one of the important factors influencing the progression of lymph node metastasis [9]. Liu et al. identified a heterogeneous cluster of cells, EDC, in axillary lymph node metastatic tissues from BC patients. Pseudotime analysis revealed that the expression of cell adhesion and migration-related genes (*CLDN3* and *F11R*) in EDC clusters showed a pattern of initial downregulation followed by upregulation during the process from in situ tissue to axillary lymph nodes. This process is similar to the epithelial-mesenchymal transition (EMT) that occurs during the metastasis of tumor cells. At the same time, marker genes related to the OXPHOS pathway (*COX6C*, *DHRS2*, *ATP5MC2*, and *NDUFB4*) showed a pattern of initial upregulation followed by downregulation, while marker genes in the glycolysis pathway (*GAPDH*, *LDHA*, *PKM*, and *PGK1*) showed a pattern of initial downregulation followed by upregulation during early dissemination of malignant epithelial cells. In other words, after EDC separates from the primary tumor, its metabolic characteristics switch from glycolysis to OXPHOS. Once the metastatic cells have seeded, the metabolic pattern returns to the glycolysis mode that promotes cell proliferation [10]. Coincidentally, this point was also confirmed by the research of Davis et al. [11]. Survival analysis based on TCGA and METABRIC datasets showed that the upregulation of OXPHOS is closely related to the poor prognosis of BC patients. Cellular experiments have confirmed that after the key OXPHOS genes *COX6C* and *DHRS2* were knocked out, the proliferation and metastasis behaviors of BC were significantly restricted. In addition, the results of cell–cell communication analysis suggest that the connection between EDC and other cells in the TME is related to tumor evasion [10].

Worth mentioning, by using spatial transcriptomics (ST), Liu et al. found that EDC clusters are located at the edge of the tumor tissue, which makes it easier for EDC clusters to fall off from the tumor tissue, thereby disseminating to the metastatic lesions. This further confirmed the close relation of EDC clusters to lymph node metastasis in BC [10]. Davis et al. [11] used a highly selective OXPHOS inhibitor "oligomycin" in a TNBC mouse model, and after inhibiting the OXPHOS process of BC cells, they found a significant reduction in the likelihood of developing lung metastases.

In summary, the energy conversion from OXPHOS to glycolysis in EDC clusters may be an important factor promoting the early metastasis of BC to lymph nodes, and key genes and inhibitors of the OXPHOS pathway may be potential targets and candidate drugs for the treatment of BC patients.

### *HMGA1* high-tumor stem cell subgroup

*CD44* and *HMGA1* are well-known markers of cancer stem cells (CSCs) [12]. Nakayama et al. found in axillary lymph node metastatic tissues of BC that, unlike *CD44* which is highly expressed in all CSCs, *HMGA1* is only highly expressed in certain specific CSC subgroups, and based on this, Nakayama et al. [13] named it the "HMGA1 high-tumor stem cell subgroup". Differential gene analysis showed that the HMGA1 high-tumor stem cell subgroup specifically expresses characteristic genes such as *TMSB10* (encoding thymosin β10, closely related to the occurrence and development of various solid tumors [14]), *CTSD* (encoding cathepsin D, a star marker for poor prognosis in BC [15]), and *LGALS1* (encoding soluble galectin-1, promoting the formation of inhibitory tumor immune microenvironment [16]), suggesting it may be a risk factor for poor prognosis in BC. Furthermore, functional enrichment analysis showed that the differential gene set of the *HMGA1* high-tumor stem cell subgroup is significantly enriched in biological processes related to ribosome synthesis and transcription activation response RNA binding protein complex formation, the upregulation of these biological processes is considered to promote distant metastasis of BC [17]. At the same time, in combination with ST, researchers found that compared to the HMGA1 high-tumor stem cell subgroup in primary tumors, the *HMGA1* high-tumor stem cell subgroup in lymph node metastasis has stronger proliferative potential, suggesting that a small number of *HMGA1*-high tumor stem cells that spread to lymph nodes can form lymph node metastasis through massive proliferation [13]. As a characteristic molecule of the *HMGA1* high-tumor stem cell subgroup—"HMGA1", a transcription factor, it regulates the transcription of target genes by altering chromatin structure and interacting with other transcription factors [18]. Previous studies have shown that HMGA1 can promote the binding of EZH2 to cancer cell DNA, promote the formation of cytoplasmic chromatin fragments (CCF), and activate the cGAS-STING pathway, which can promote the production of inflammatory factors and the metastasis of BC [19, 20]. Sgubin M et al. demonstrated in a mouse model that, after silencing *HMGA1*, the metastatic activity of BC cells significantly decreased; this process was realized through the HMGA1/p27/stathmin axis, and the research results showed that after inducing HMGA1 depletion, the expression and activity of stathmin (an unstable phosphoprotein, which is overexpressed in metastatic tumors [21]) on microtubules were downregulated, leading to a decrease in microtubule dynamics, resulting in reduced mobility of TNBC cells and thus reduced tumor metastasis. In addition, Sgubin et al. [22] also found that HMGA1 plays an important role in tumor drug resistance, and after silencing *HMGA1*, the sensitivity of BC cells to “paclitaxel” (paclitaxel is a drug used for TNBC treatment) increased. Wang et al. [23] also found that in BC patients with high *HMGA1* expression, their survival rate is lower.

In conclusion, the existence of the HMGA1 high-tumor stem cell subgroup is closely related to tumor metastasis and drug resistance.

### ***CXCL14***^+^ tumor cell subgroup

Xu et al. identified a tumor cell subgroup related to metastasis in lymph node metastasis tissues of BC "*CXCL14*^+^ tumor cell subgroup (*CXCL14*^+^CancerCells)". This subgroup specifically overexpressed genes related to BC lymph node metastasis, such as *CXCL14, KAT6B, CARTPA, UGT2B11, COX6C,* and *MT-TV*. At the same time, GO enrichment analysis shows that process such as amino acid transport, RNA polymerase 1 complex, and activity were only enriched in the *CXCL14*^+^ tumor cell subgroup and not in other tumor subgroups, suggesting that this subgroup is actively proliferating. Further pseudotime analysis indicated that the *CXCL14*^+^ tumor cell subgroup was mainly located at the end of the differentiation trajectory and was mainly present in BC lymph node metastasis tissues. Analysis using the Oncomine database showed that *CXCL14*^+^ had a higher expression level in BC patients with lymph node metastasis, suggesting that this subgroup is closely related to BC metastasis [24]. Interestingly, through cell communication analysis, it was found that the highly expressed *CD47* in the *CXCL14*^+^ tumor cell subgroup can bind to SIRPA on the macrophage membrane, delivering a "self-signal" that prevents the subgroup from being phagocytosed by macrophages [25]. This might be the reason why the *CXCL14*^+^ Cancer Cells evade innate immune surveillance and avoid being cleared by phagocytosis.

In summary, the *CXCL14*^+^ tumor cell subgroup is mainly present in BC lymph node metastasis tissues and is closely related to BC metastasis. However, its further role still needs to be verified by more experiments.

### Subgroup of osteoclast-like giant cells (OGCs)

BC with OGCs is a rare subtype of BC. When performing single-cell sequencing analysis on axillary lymph node metastasis tissues of BC, Xu et al. [26] identified a subgroup with OGCs marker genes (*ACP5, MMP9, CST3, CD68*). This cell type is extremely rare in BC (< 2%) and OGCs primarily express the M2-macrophage phenotype rather than the M1-macrophage phenotype [27]. Researchers have observed that, in hepatocellular carcinoma and some BCs, the presence of OGCs indicates an invasive clinical course [28, 29]; Hatano et al. [30] found that some OGCs in the TME can promote tumor growth and lymphatic vessel formation by secreting VEGF-C. Therefore, the presence of OGCs can promote BC metastasis. However, there is currently no clear understanding of OGCs in BC.

Regarding the origin of OGCs, one hypothesis suggests that cancer cells secrete vascular endothelial growth factor, promoting macrophage angiogenesis and migration into the tumor, then inducing monocytes to fuse with stromal cells to become OGCs [31]. As for the function of OGCs in BC, further, GO analysis by Xu et al. [26] showed that its most enriched biological process is oxidative phosphorylation. It is noteworthy that past research has confirmed that the RANK-L/RANK axis is a key participant in breast development. In mice, RANK-L, influenced by progesterone, is expressed by luminal cells and can act on breast stem cells in a paracrine manner to promote their proliferation and resistance to apoptosis [32, 33]. Meanwhile, Cyrta et al. [34] have proven that almost all BC tumor cells containing OGCs express the RANK-L protein, while BC tumor cells without OGCs do not express the RANK-L protein.

In addition, the RANK-L/RANK axis is also related to breast tumorigenesis and epithelial-mesenchymal transition. In mouse models, RANK-L inhibition has been proven to reduce the formation of BC [33, 35, 36]. More excitingly, Turgeman et al. [37] reported a case of successful treatment of a patient with OGCs in BC bone metastasis using “denosumab”. After receiving subcutaneous injections of 120 mg denosumab for 8 months, CT scans showed that her bone metastases had almost completely disappeared. Therefore, for patients with OGCs, the RANK-L targeted drug "denosumab" can be used for treatment. This also indirectly indicates that the presence of OGCs is related to BC metastasis.

Furthermore, the use of ST further reveals its relationship with the location of tumor cells. Xu et al. [26] found that OGCs are dispersed in BC tumor tissues, not clustered together, and they can secrete pro-tumor factors themselves, which further suggests that the growth or survival of tumor cells in metastatic lesions depends on OGCs [38].

In conclusion, there is a more detailed description of the function and origin of OGCs, as well as their spatial distribution characteristics in tumor tissues. Using the RANK-L targeted drug "denosumab" may achieve good therapeutic effects for BC patients with OGCs in metastatic lesions.

## Special cell subgroups related to metastasis in distant metastasis of breast cancer

Compared with lymph node metastasis of BC, patients with distant metastasis of BC have extremely poor prognoses and often lose the opportunity for surgery [39]. Although in recent years, systemic therapies such as immune checkpoint blockade have been proven to show some prospects in the treatment of distant metastasis of BC, their therapeutic effects still have high heterogeneity [40, 41]. Therefore, the identification of special tumor cell subgroups in distant metastasis of BC through single-cell sequencing can help reveal its drug resistance mechanisms and develop new immunotherapy targets.

### Tumor stem cell subgroup in lung metastasis—lung L3

When analyzing tumor cells in lung metastasis tissue of mouse BC through single-cell sequencing, a study identified a *CD44*^Low^ CSC (Cancer Stem Cell) subgroup "Lung L3" [42]. Some stem cell-related genes *ALDH1A1, ABCG2,* and *SNAI2* were highly expressed in L3, but some other stem cell-related genes (*ME1, HIF1A*) were downregulated. At the same time, the marker gene *CD24A* of weakened cell proliferation and stem potential was also increased in Lung L3 cells. It is worth noting that cancer dormancy is an obstacle in BC research and treatment [43], and some dormant BC cells have genomic features similar to CSC [44, 45]. However, as a subgroup of CSC, L3 does not express genes related to cancer dormancy (*FN1, CD47, and THBS1*); therefore, L3 is a smaller, independent cluster that expresses high levels of stem cell markers while lacking the expression of cancer dormancy marker genes.

### Subgroup of ***TREM2***^+^ macrophages in bone marrow metastasis

Molgora et al. [46] have identified a group of tumor-associated macrophages expressing high levels of *TREM2* through single-cell sequencing of bone marrow metastasis tissue in mouse models of BC. Further experiments showed that *TREM2*^+^ tumor-associated macrophages exist in various types of tumors and co-express *CD68, CD163, and CSF1R*. TREM2 is a cell surface receptor responsible for transmitting intracellular signals to maintain microglial response. It can bind lipids and transmit intracellular signals via the adapter DAP12 [47]. Further experiments showed that the lack of TREM2 can inhibit the growth of tumor-infiltrating macrophages, reduce the number of *CX3CR1*^+^ and *MRC1*^+^ macrophage subgroups, and enhance the reactivity of CD8^+^ T cells to anti-PD-L1, thereby remodeling the TME in mouse bone marrow metastasis [47].

The above findings suggest that the TREM2^+^ macrophage subgroup may play an important role in tumor development and metastasis and could serve as a new target for combined immunotherapy to enhance the efficacy of anti-PD-L1 treatment.

### Dormant cancer cells (DTCs)

The presence of DTCs means that BC patients may relapse after treatment during the early stages of cancer; however, due to the low numbers of DTCs, they are difficult to detect, making it challenging to predict which patients are prone to tumor recurrence. Ren et al. used the advantage of single-cell sequencing to isolate DTCs from a mouse model and conducted an analysis. The analysis showed that regardless of the metastatic tissue, DTCs express a similar and unique set of gene features (*Cfh, Gas6, Mme, Ogn, Postn, Pdgfrb, and Aldh1a1*). Additionally, in dormant BC cells in the bone and lung, genes like *Cfh, Gas6, Mme,* and *Ogn* were highly expressed. Gene ontology enrichment analysis of differentially expressed genes in dormant cancer cells showed that genes involved in immune system processes (such as inflammatory response and immune response) are enriched in DTCs. Furthermore, Ren et al. [48] utilized public database to analyze dormancy-related genes. The results indicated that patients with high expression levels of these genes showed a significant survival advantage compared to those with lower expression levels. Similarly, Bidard et al. [49] also found that DTCs in the bone metastatic tumor tissue of BC can be used to predict cancer recurrence in BC patients. These findings suggest that although DTCs might be activated and responsible for cancer recurrence under certain circumstances, breast cancer with high dormancy-related gene signatures is more likely to maintain a dormant state rather than triggering cancer recurrence, thus leading to an overall better prognosis [48]. Bone marrow colonization and metastasis are the reasons for ER^+^ BC recurrence, a process mainly regulated by hormones (and the microenvironment). Anti-estrogen therapy is an effective method for treating human BC but cannot completely cure BC. To understand the mechanism, Hong et al. [50] used single-cell sequencing to find that DTCs are prevalent in BC tumor tissue during anti-estrogen therapy, which can explain why BC recurs after anti-estrogen therapy from a cellular perspective. In summary, the presence of DTCs increases the chances of BC recurrence and may affect the effectiveness of anti-estrogen therapy.

There are also many studies on the impact of DTCs on BC metastasis. Among them, Er EE and others found that the adhesion molecule L1CAM on DTCs can mediate entry into the perivascular niche, driving the transcription program to awaken DTCs, leading to the initiation of metastasis [51]. Other research indicates that similar to benign and malignant hematopoietic cell mechanisms, BC DTCs enter the cerebrospinal fluid through blood vessels and express inflammatory molecules E-selectin and SDF-1 in mouse and human samples [52]. Experiments have shown that in mouse models of BC treated with E-selectin inhibitors, the DTCs in their bone marrow are significantly reduced [52]. In summary, DTCs are a rare but significant cell type in BC metastatic tissue in terms of tumor recurrence and metastasis. Under normal conditions, it remains in a dormant state, much like a seed, and exhibits high expression of dormancy-related genes. However, in special circumstances, DTCs are activated, and the dormant state is lifted, making it more susceptible to metastasis and recurrence.

## Summary and outlook

Single-cell sequencing technology has identified cell subgroups related to BC metastasis during the process from the start point of metastasis (primary tumor) to the endpoint (target tissue). However, there is a lack of a systematic summary of these cell subgroups in the literature. This paper is the first to summarize cell subgroups related to BC metastasis at the single-cell level. It introduces cell subgroups related to BC metastasis in different tissues such as primary tumors, axillary lymph nodes, distant lymph nodes, bone marrow, lungs (Fig. 1 and Table 1) and describes them from the perspectives of their molecular features, biological functions, cell communication, and evolution trajectories. Besides, in combination with ST and experiments, their impact on BC metastasis can be further verified. Finally, based on the characteristics of each subgroup, drugs or methods are sought to predict, prevent, and suppress the metastasis of BC, which is of great significance for improving the prognosis of BC patients. There is still room for improvement in the use of single-cell transcriptomics for the study of BC metastasis today: 1)Overcoming sampling restrictions: Single-cell transcriptomics typically requires cell samples from patient tissues or blood. Obtaining a sufficient quantity and high-quality samples can be challenging, especially for rare metastatic foci. With continuous technological advancement, we expect higher resolution, sensitivity, and throughput single-cell transcriptomics techniques. This will allow researchers to comprehensively and accurately describe cellular heterogeneity and signaling pathway changes in the process of BC metastasis, even with a very small amount of tissue; 2) Integration of multi-omics data: Single-cell transcriptomics is often combined with other omics techniques (such as single-cell proteomics, single-cell epigenetics, ST) to obtain more comprehensive molecular information. Integrating single-cell transcriptomics data with other omics data can provide a deeper understanding of the mechanism of BC metastasis and discover new treatment targets. 3) Improvement in data analysis and interpretation methods: As the amount of single-cell transcriptomics data increases, data analysis and interpretation methods will also improve. New algorithms and tools will be developed to handle complex single-cell data and provide more accurate and efficient data interpretation and result presentation. 4) Personalized treatment strategies: Single-cell transcriptomics can help identify metastasis-related cell subgroups and key signaling pathways driving metastasis in BC patients and help discover new metastasis markers and potential targets for targeted treatment. This will provide more accurate information for the formulation of personalized treatment strategies, help optimize treatment plans, and improve patient prognosis. 5) Clinical applications: As the technology matures and data accumulates, the clinical application of single-cell transcriptomics in BC metastasis is expected to expand. Single-cell transcriptomics data could become an important basis for auxiliary diagnosis, prognosis assessment, and treatment decisions, helping doctors provide more personalized treatment plans for patients.

**Table 1:** Sample, molecular characteristics, function, and reference of cell subpopulations

Cell subpopulations	Sample	Molecular characteristics	Function	References
ARTN + L1CAM + epi_tum1	Primary Cancer	ARTN, L1CAM	Promotes angiogenesis and metastasis	Shi et al
HMGA1-high Cancer Stem Cell	Axillary lymph	HMGA1	Promote lymph node metastasis	Nakayama et al
Early-disseminated Cancer Cell	Axillary lymph	COX6C, DHRS2	Metastasis of energy metabolism	Liu et al
CXCL14 + Cancer Cell	Lymph node	CXCL14, KAT6B	Evade immune surveillance during metastasis	Xu et al
Osteoclast-like giant Cell	Lymph node	MMP9, CST3, CD68	Promote the growth of metastatic cells	Xu et al
Lung L3 Cancer Stem Cell	Lung	ALDH1A1, ABCG2	Promote lung metastasis	Xu et al
TREM2 + Macrophage	Bone marrow	TREM2, CD68	Promote bone marrow metastasis	Molgora et al
Dormant Cancer Cell	Tissue	CFH, GAS6	Promotes bone/cerebrospinal fluid metastasis	Bidard et al

In addition, biases that may have led to the emergence of specific or rare subgroups mentioned in the text could have occurred during processes such as dissociation and bioinformatics analysis. Therefore, cross-study validations among different studies are crucial. Researchers can utilize single-cell databases to further verify the characteristic genes, cellular functions, differentiation trajectories, and cell communication of identified rare subgroups, which can provide stronger evidence for the existence of these specific subgroups discussed in the article.

For rare subgroups, they may only be present in a small number of patients or at specific stages, making their validation more complex. Single-nucleus RNA sequencing (snRNA-seq) is better suited for studying such cell subgroups. It can be applied to frozen tissues, where cellular transcription activities have been suppressed and fixed, thus eliminating transcriptional state changes. This enhances the authenticity of the results. Additionally, when cells are mechanically or chemically disrupted in a frozen state, it avoids the dissociation biases introduced by enzymatic methods, resulting in a more comprehensive representation of cell types.

Taken together, BC can metastasize to many parts of the body, but there are still few articles using single-cell sequencing technology to study BC metastasis. With the continuous development and improvement of technology, we believe that single-cell transcriptomics will further play a vital role in cancer metastasis research.

## Data Availability

Not applicable.

## Abbreviations

BC: Breast cancer
TNBC: Triple-negative breast cancer
TME: Tumor microenvironment
ER^+^: Estrogen receptor-positive
HIF1A: Hypoxia-inducible factor 1 subunit alpha
ST: Spatial transcriptomics
CSCs: Cancer stem cells
CCF: Cytoplasmic chromatin fragments
EDC: Early Dissemination of Cancer cells
HCC: Hepatocellular carcinoma
EMT: Epithelial-mesenchymal transition
OGCs: Osteoclast-like giant cells
DTCs: Dormant cancer cells
